# Supplementary material for: Computational modeling of seizure spread on a cortical surface
Source: J Comput Neurosci. 2021 Oct 23;50(1):17–31. doi: 10.1007/s10827-021-00802-8 (PMC8818012; doi:10.1007/s10827-021-00802-8)
Supplement: Supplementary file 1 — Supplementary file1 (DOCX 256 KB) [file 10827_2021_802_MOESM1_ESM.pdf]

# Supplementary Information: Computational modeling of seizure spread on a cortical surface

Viktor Sip[1] Maxime Guye[2,3] Fabrice Bartolomei[1,4] Viktor Jirsa[1*]

**1** Aix Marseille Univ, INSERM, INS, Inst Neurosci Syst, Marseille, France
**2** Aix Marseille Univ, CNRS, CRMBM, Marseille, France
**3** Assistance Publique - Hôpitaux de Marseille, Hôpital de la Timone, CEMEREM, Pôle d'Imagerie Médicale, CHU, Marseille, France
**4** Assistance Publique - Hôpitaux de Marseille, Hôpital de la Timone, Service de Neurophysiologie Clinique, CHU, Marseille, France

* viktor.jirsa@univ-amu.fr

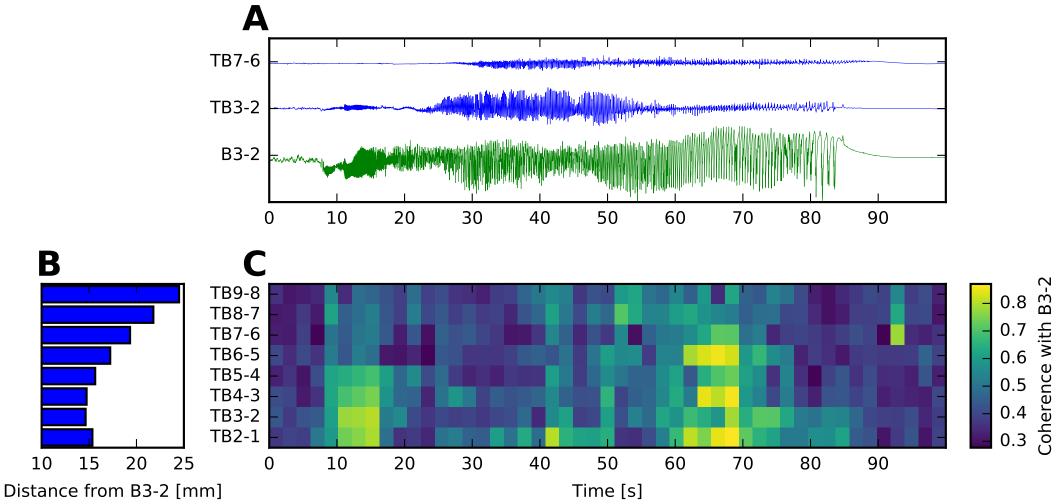


**Figure S1.** Coherence indicates that the initial oscillatory activity on the contacts of the TB electrode are due to the volume conduction. (A) Selected bipolar traces. On B3-2 and TB3-2, a fast (~20 Hz) oscillatory activity appears between 8 and 18 s, with high amplitudes at B3-2 and lower at TB3-2. (B) Euclidean distance of a midpoint between two neighboring contacts to the midpoint B3-2. (C) Average coherence with B3-2 in a frequency band [1, 30] Hz. At 8 s, high coherence values are appears on channels TB2-1 to TB5-4, disappearing again after 18 s. In this period the coherence is highest at TB3-2, which is nearest to B3-2, and decays with increasing distance. High values of coherence reappear later (60 - 70 s) as the seizure progresses, however, without the clear dependence on the distance from B3-2. In our view, this indicates that the initial oscillations on all electrodes reflect the activity at right hippocampus, where B3-2 is placed, and are seen only due to the volume conduction. The later oscillations on the other hand reflect the source activity close to the contacts, and are coherent due to the source synchronization.

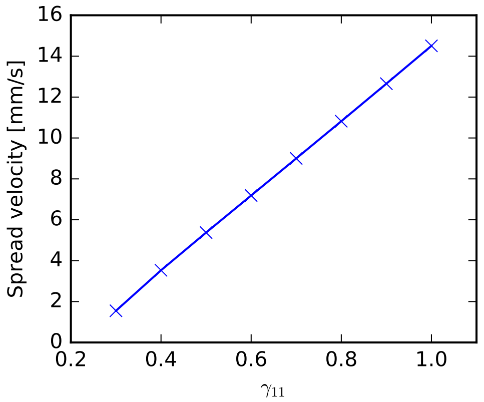


**Figure S2.** Dependence of the seizure spread velocity on the coupling strength $\gamma_{11}$. The relation was determined by numerical simulations of a seizure spread on a 2D rectangular patch with varying $\gamma_{11}$ (see Methods). For $\gamma_{11} < 0.3$ the seizure failed to propagate from the epileptogenic zone.

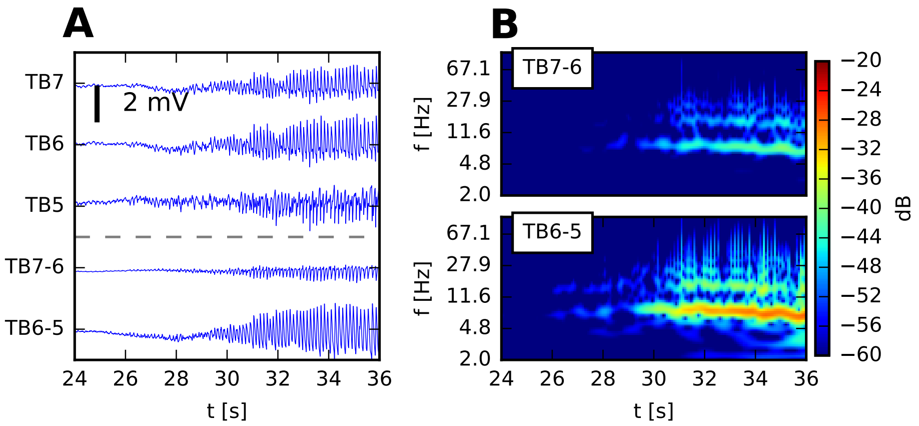


**Figure S3.** Spectral signatures of the recorded SEEG signals. Cf. Fig. 8 in the main text. (A) Monopolar and bipolar view of seizure onset on selected contacts. (B) Spectrogram of the two bipolar signals. $1/f$ normalization (spectral flattening) was applied to the spectrogram.
